# T cell-derived lymphotoxin limits Th1 response during HSV-1 infection

**DOI:** 10.1038/s41598-018-36012-z

**Published:** 2018-12-07

**Authors:** Kaiting Yang, Yong Liang, Zhichen Sun, Longchao Liu, Jing Liao, Hairong Xu, Mingzhao Zhu, Yang-Xin Fu, Hua Peng

**Affiliations:** 1 Key Laboratory of Infection and Immunity, Institute of Biophysics, Chinese Academy of Sciences, Beijing, 100101 China; 2 University of Chinese Academy of Sciences, Beijing, 100049 China; 3 Department of Pathology, University of Texas Southwestern Medical Center, Dallas, TX 75390 USA

## Abstract

Though lymphotoxin (LT) is highly expressed by type I helper T (Th1) cells, its contribution to CD4^+^ T cell differentiation during infections and diseases remains a mystery. In HSV-1 infection, we observed that LTβR signaling is required to limit the Th1 response. Using bone marrow chimeric mice, mixed-T-cell chimeric mice, and LTβR *in vivo* blockades, we unexpectedly observed that LT, especially T cell-derived LT, played an indispensable role in limiting the Th1 response. The LTβR-Ig blockade promoted the Th1 response by increasing infiltration of monocytes and monocyte-derived DCs and up-regulating IL-12 secretion in the lymphoid environment. Our findings identified a novel role for T cell-derived LT in manipulating Th1 differentiation.

## Introduction

During the adaptive immune response, activated CD4^+^ T cells will expand and differentiate into several subpopulations, including Th1, Th2, Th17, Treg and T follicular helper (Tfh) cells, under regulation of distinct sets of cytokines and transcriptional factors^1^. Lymphotoxin (LT, in the form of membrane heterotrimer (LTα_1_β_2_) or secreted homotrimer (LTα_3_), is expressed on activated B and T cells, especially expressed constitutively on Th1 cells but not Th2 cells^2^. LTα_3_ binds to TNFR1 and TNFR2^3^. The receptor for LTα_1_β_2_, Lymphotoxin-beta-Receptor (LTβR), is expressed on follicular dendritic cells (FDCs), DCs, macrophages and stromal cells^4^. Activation of the LTβR pathway stimulates the expression of pro-inflammatory mediators, adhesion molecules^5^ and lymphocyte-recruiting chemokines, such as CCL19/CCL21 and CXCL13. Chemokine gradients help to define the B and T cell zones, thereby establishing the primary and secondary lymphoid structures^6–8^. Beyond that, the LT-LTβR signaling also helps to mediate the adaptive immune response. LT expression on activated helper T cells plays a critical role in mediating full DC maturation, indispensable for optimal CTL response^9^. The lymphotoxin-induced signaling deficiency leads to an increased susceptibility to some bacterial infections in association with the impaired Th1 response^10,11^. As a hallmark molecule of Th1 cells, LT is thought to facilitate Th1 differentiation by supporting the lymphoid tissue development^12^, but whether LT promotes Th1 differentiation in viral infections has not been proven experimentally.

HSV-1 (herpes simplex virus type 1) is a double-stranded DNA virus that can cause acute and latent infections in human beings, often used as an acute Th1-biased viral infection model in mice^13,14^. Our previous finding suggests that Tfh-like cells will acquire a Th1-like feature in the LTα_1_β_2_-LTβR signaling deficient environment post HSV-1 foot-pad infection^15^, based on which, we suppose that the LT-LTβR signaling may somehow limit Th1 over-differentiation. In this study, an LTβR *in vivo* blockade (LTβR-Ig) was employed to investigate how the LTα_1_β_2_-LTβR induced signaling is involved in controlling the Th1 differentiation within well-established lymphoid structures during HSV-1 infection. We revealed that T cell-derived LTα_1_β_2_ could unexpectedly limit the Th1 response by constricting expression of IL-12 from monocyte-derived cells.

## Materials and Methods

### Mice

The mice used in this work all were on a C57BL/6 background. Wild type C57BL/6 mice were purchased from Beijing Vital River Co., Ltd. *Ltb*^−/−^, *Lta*^−/−^, *Light*^−/−^, *Tcra*^−/−^, μMT, OT-II transgenic, *Lta*-deficient OT-II transgenic and LysM^*ΔLtbr*^ mice were bred and housed under specific pathogen–free (SPF) conditions. *Tcra*^−/−^ and OT-II transgenic mice were obtained from The Jackson Laboratory. Mice were used at 6–10 weeks of age. Animal care and experiments were performed in accordance with the guidelines of the Institute of Biophysics, Chinese Academy of Sciences, using protocols approved by the Institutional Laboratory Animal Care and Use Committee, including any relevant details.

### Foot-pad infection and vaccination model

HSV-1 (strain 17) was kindly provided by Dr. Thomas Kristie, LVD/NIAID/NIH, and amplified with *vero* cells (ATCC) purified through sucrose-dextran gradient centrifuge^16^. A total of 5 × 10^7^ pfu of HSV-1 in 50 μl of PBS was subcutaneously injected into the foot-pad of mice after anesthesia. In the Heat-iHSV (heat-inactivated HSV-1) infection model, 5 × 10^7^ pfu of HSV-1 was heat-inactivated at 60 °C for 30 min and injected into the mouse foot-pad. In an OVA-CpG foot-pad vaccination model, mixture of 100 μg of OVA and 50 μg of CpG-1826 was injected into the foot-pad.

### *In vivo* blockade of LTβR signaling or cytokines

LTβR-Ig was i.p. administered, 100 μg/mouse one day before HSV-1 infection^17^. Control mice were injected with the same volume of the carrier only (PBS) or human IgG (100 μg/mouse). Anti-IFNγ (XMG1.2, Bioxell, US), anti-IL-12p75 (R5-9A2, Bioxell, US) or control rat IgG (Biogen, US) was i.p. administered, 500 μg/mouse every third day, three times in total, as described before^18,19^.

### Measurement of IFNγ-secreting CD4^+^ T cells

Cells isolated from the draining LNs (popliteal LN and inguinal LN) were re-suspended in R10 medium (RPMI1640 supplemented with 10% FBS, 2 mM L-glutamine, 100 U/mL penicillin, and 100 μg/mL streptomycin). CD8^−^ cells were purified using an EasySep Biotin Selection Kit (STEMCELL, Canada), and a total of 2 × 10^5^ purified cells were stimulated with Heat-iHSV (heat-inactivated at 60 °C for 30 min, MOI = 10) for 24–48 hrs. The number of IFNγ−secreting CD4^+^ cells was determined by an IFNγ ELISPOT Assay Kit (BD Biosciences, US). Spots were captured and calculated with the ImmunoSpot Analyzer (CTL, US).

### Detection of cytokines

Lymph nodes were homogenized and centrifuged at 12, 000 × g, 10 min, for supernatant collection. Protease Inhibitor Cocktail (100× , CWBio, China) was added during supernatant collection. IFN-γ, IL-12p70 and MCP-1 levels in the supernatant were detected by mouse Inflammation CBA assay (BD Biosciences, US).

### mRNA and DNA detection

RNA was extracted using the RNeasy Plus Universal Micro kit (Qiagen, US). The quality and quantity of the total RNA were assessed with Nanodrop spectrophotometer (ND 2000C; Thermo Fisher Scientific, US). cDNA was reverse-transcribed using a First Strand cDNA Synthesis Kit (Thermo Scientific, US). Real-time RT-PCR was performed using an ABI7500 (Applied Biosystems, US). cDNA was amplified using SYBR Premix Ex Taq^TM^ mix (Takara, Japan). HSV DNA was extracted using the TIANamp DNA kit (TIANGEN, China). HSV DNA level was normalized to GAPDH and murine gene expression was normalized to *β-actin* and calculated using 7500 software v2.0.6 (Applied Biosystems, US). Primers used are listed: HSV-gE: forward, 5′-GGGAGCACCACATAACCGACC-3′; reverse, 5′-GGCAAAGTCAACACAACAACGC-3′; GAPDH: forward, 5′-CGGACTGCAGCCCTCCC-3′; reverse, 5′-CCTTCCCAGTTTCCGACTGTCC-3′; *Tbx21*: forward, 5′-AGCAAGGACGGCGAATGTT-3′; reverse, 5′-GGGTGGACATATAAGCGGTTC-3′; *Ifng*: forward, 5′-CTCTGAGACAATGAACGCTACA-3′; reverse, 5′-TCTTCCACATCTATGCCACTT-3′; *Gata3*: forward, 5′-CTCGGCCATTCGTACATGGAA-3′; reverse, 5′-GGATACCTCTGCACCGTAGC-3′; *Il4*: forward, 5′-GGTCTCAACCCCCAGCTAGT-3′; reverse, 5′-GCCGATGATCTCTCTCAAGTGAT-3′; *Bcl6*: forward, 5′-CCGGCACGCTAGTGATGTT-3′; reverse, 5′-TGTCTTATGGGCTCTAAACTGCT-3′; *Il21*: forward, 5′-GGACCCTTGTCTGTCTGGTAG -3′; reverse, 5′-TGTGGAGCTGATAGAAGTTCAGG-3′; *β-actin*: forward, 5′-ACACCCGCCACCAGTTCGC-3′; reverse, 5′-ATGGGGTACTTCAGGGTCAGGGTCAGGATA-3′.

### Surface and intracellular staining

Draining lymph nodes were digested with 1 mg/mL collagenase IV (Sigma-Aldrich, US) and 200 μg/mL DNaseI (Sigma-Aldrich, US) at 37 °C for 30 minutes. Flow cytometry data were acquired on an LSRFortess (BD, US) with FACSDiva software (BD, US). Data were analyzed using FlowJo software (Tree Star, Ashland, USA). Dead cells were excluded by LIVE/DEAD^®^ Fixable Yellow Dead Cell Stain Kit (Invitrogen, US). The antibodies used were: anti-CD11b (M1/70), anti-CD4 (RM4-5), anti-CD8α (53–6.7) and anti-Tbet (eBio4B10) from eBioscience (US); anti-CD11c (N418), anti-CD8α (53–6.7), anti-Ly6C (HK1.4) and anti-MHCII (M5/114.15.2) from BioLegend (US).

### Cell sorting

Draining lymph nodes were digested with 1 mg/mL collagenase IV (Sigma-Aldrich, US) and 200 μg/mL DNaseI (Sigma-Aldrich, US) at 37 °C for 30 minutes. CD4^+ ^T cells (CD3^+^CD4^+^), monocytes (CD11c^−^CD11b^+^Ly6C^hi^MHC-II^+^), moDCs (CD11c^+^CD11b^+^Ly6C^hi^MHC-II^+^) and non-monocyte-derived DCs (CD11c^+^Ly6C^−^MHC-II^+^, used as control) were sorted on an AriaII (BD, US). Purified cells were checked using LSRFortess (BD, US) with FACSDiva software (BD, US). Purity of the indicated cell population was more than 90%. The antibodies used were: anti-CD3ε (145-2C11), anti-CD4 (RM4-5), anti-CD11b (M1/70) from eBioscience (US); anti-CD11c (N418), anti-CD8α (53-6.7), anti-Ly6C (HK1.4) and anti-MHCII (M5/114.15.2) from BioLegend (US).

### Generation of bone marrow chimeras

Bone marrow cells (2 × 10^6^) from the indicated donors were transferred intravenously into each recipient mouse, which had been lethally irradiated with a single dose of 1000 rad. Donor-derived cells can be found in the peripheral blood or primary and secondary lymphoid organs of the recipient mice after reconstitution^20^. The bone-marrow chimeric mice were infected after an approximately 8-week reconstitution after check.

### Statistical Analysis

Data were shown by GraphPad Prism software (version 7.0, US). For XY curves, statistical analyses were performed using the two-way ANOVA multiple comparisons, with data shown as the mean ± SEM. For histograms, statistical analyses were performed using a two-tailed unpaired Student’s *t* test unless stated otherwise, with data shown as the mean ± SEM. Differences with a *P*-value < 0.05 were considered significant. n.s., not significant; *P < 0.05; **P < 0.01; ***P < 0.001, ****P < 0.0001.

## Results

### LTα_1_β_2_-LTβR signaling is required to limit an excessive anti-HSV-1 Th1 response

**Figure 1:**
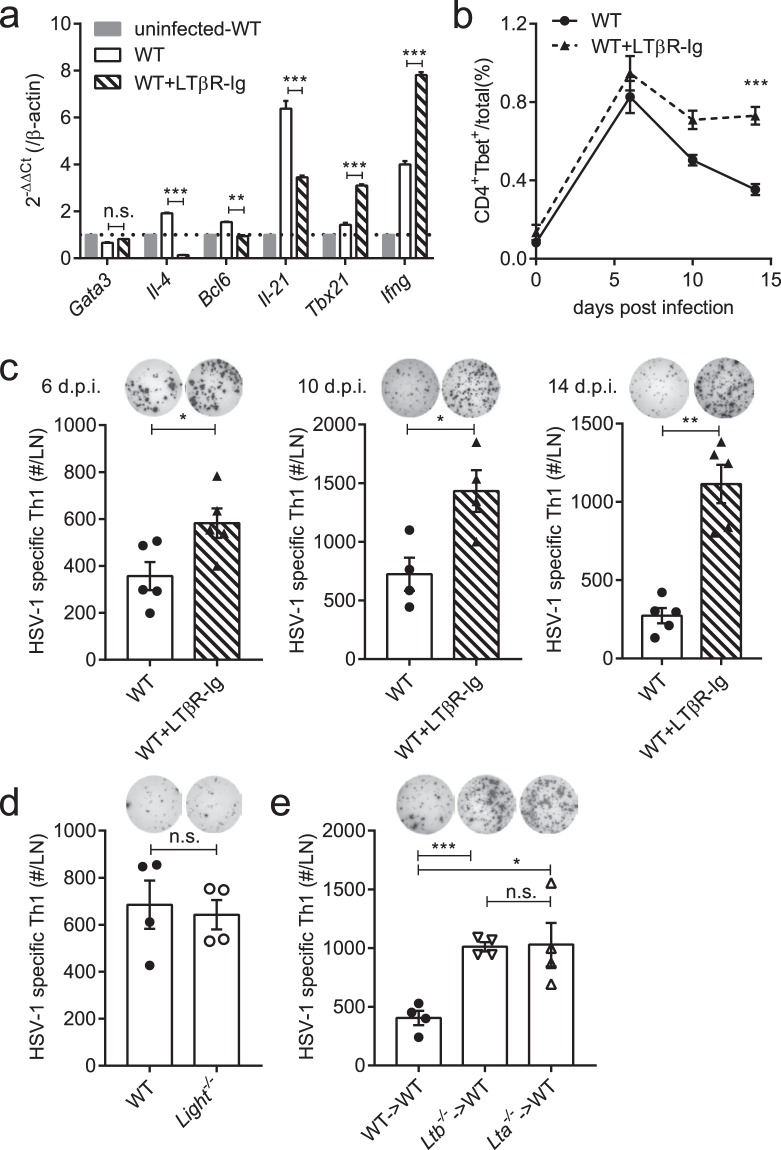
LT-LTβR signaling deficiency enhances the anti-HSV-1 Th1 response. (**a**) Expression of the Th1/Th2/Tfh-related transcriptional factors and cytokines in purified CD4^+^ T cells on day 4 p.i. in the popliteal LNs tested by real-time qPCR (n = 3/group). (**b)** Percentages of CD4^+^Tbet^+^ cells in total cells in the draining LNs (popliteal LN and inguinal LN) from WT mice (solid line) and LTβR-Ig-treated mice (dotted line) (n = 5/group). (**c)** The Th1 response of LTβR-Ig-treated mice on 6 d.p.i. (n = 5/group), 10 d.p.i. (n = 4/group) and 14 d.p.i. (n = 5/group), including immunospots and absolute numbers of IFNγ-secreting CD4^+^ cells per LN. (**d)** The Th1 response of *Light*^−/−^ mice on day 14 p.i. (n = 4/group), including immunospots and absolute numbers of IFNγ-secreting CD4^+^ cells per LN. (**e)** The Th1 response of *Lta*^−/−^ → WT and *Ltb*^−/−^ → WT bone marrow chimeric mice on day 14 p.i. (n = 4/group), including immunospots and absolute numbers of IFNγ-secreting CD4^+^ cells per LN. Data are representative of three independent experiments, shown as mean ± SEM. n.s., not significant; *P < 0.05; **P < 0.01; ***P < 0.001. (Two-way ANOVA multiple comparisons for **b**).

Differentiation of helper T cells is controlled by the LTβR mediated signaling but may differ under specific infections or diseases. To describe the differentiation of helper T cells after HSV-1 infection, mRNA from CD4^+^ T cells in the popliteal lymph nodes was analyzed on day 4 post HSV-1 foot-pad infection. In CD4^+^ T cells purified from LTβR-Ig-treated mice, lower expression of Th2 signature genes (*Il-4*) and Tfh signature genes (*Bcl6*, *Il-21*)^21,22^ was observed, while the expression of Th1 signature genes (*Tbx21*, *Ifng*)^23^ was overly up-regulated (Fig. 1a), showing an enhanced Th1-biased feature. Furthermore, during the process of helper T cell differentiation, the percentage of CD4^+^Tbet^+^ cells remained high in LTβR-Ig-treated mice but declined in wild-type (WT) mice (Fig. 1b, gating strategy shown in Supplementary Fig. 1a and number of CD4^+^Tbet^+^ cells shown in Supplementary Fig. 1b). Further, IFNγ-secreting CD4^+^ T cells were detected on day 6, day 10 and day 14 post infection (p.i.) (Fig. 1c) using an enzyme linked immunospot assay (Elispot) under *ex vivo* stimulation with heat-inactivated HSV-1 (Heat-iHSV) for 24–48 hours. Consistent with the up-regulation of Th1 signature genes and the increasing of CD4^+^Tbet^+^ cells, the anti-HSV-1 Th1 response was continuously enhanced in the LTβR-Ig-treated mice over time. The LTβR-Ig-induced prolonged increasing of Tbet^+^CD4^+^ T cells from day 6 p.i. (Fig. 1b) finally resulted in an enhanced Th1 response on day 14 p.i. These together suggest that activation of the LTβR pathway limits Th1 cell development in HSV-1 infection.

There are two ligands for activation of the LTβR pathway, LTα_1_β_2_ and LIGHT (homologous to LTα_1_β_2_). We previously found that the innate LTα_1_β_2_/LIGHT signaling could promote an HSV-1-induced inflammation in immunocompromised mice^24^. To further identify the contributing ligand for LTβR in limiting the Th1 response in a wild-type (WT) background, mice with homozygous deletion for LIGHT (*Light*^−/−^) or LTα_1_β_2_ (*Ltb*^−/−^ and *Lta*^−/−^) were used. Comparable Th1 responses were detected in *Light*^−/−^ and WT mice (Fig. 1d). However, *Lta*^−/−^ mice were reported to be more susceptible to HSV-1 infections^25^, where lack of lymph nodes (LNs) and CTL responses were thought to be the major cause. To rule out the interference from the disorganized lymphoid structures, bone marrow chimeric mice were prepared and infected. Bone marrow cells from *Ltb*^−/−^, *Lta*^−/−^ *vs*. WT mice were adoptively transferred to the WT mice before infection, abbreviated as *Ltb*^−/−^ → WT, *Lta*^−/−^ → WT, and WT → WT. *Ltb*^−/−^ → WT and *Lta*^−/−^ → WT mice both generated an enhanced anti-HSV-1 Th1 response, compared to the WT → WT mice (Fig. 1e). Together, these indicate that LTα_1_β_2_ induces a limitation of the Th1 response in HSV-1 infection.

### The LTα_1_β_2_-LTβR induced Th1 limitation is not restricted to a specific viral infection

**Figure 2:**
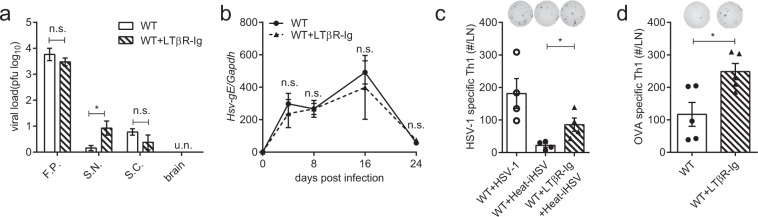
The LTβR-Ig blockade results in an enhanced Th1 response in a non-viral-replication-dependent manner. (**a)** Viral load of peripheral nervous tissues tested on day 8 p.i. (F.P.: foot-pad; S.N.: sciatic nerves; S.C.: spinal cord. n = 4/group). (**b)** HSV-gE detected in the DLN (n = 4/group). (**c)** Th1 response in the Heat-iHSV infection model on day 14 p.i. 5 × 10^7^ pfu of HSV-1 was heat-inactivated at 60 °C for 30 min and injected into the foot-pad. (n = 4/group), including immunospots and absolute numbers of IFNγ-secreting CD4^+^ cells per LN. (**d)** Th1 response in an OVA-CpG foot-pad vaccination model (100 μg of OVA and 50 μg of CpG-1826) on day 10 post immunization (n = 5/group), including immunospots and absolute numbers of IFNγ-secreting CD4^+^ cells per LN. Data are representative of three independent experiments, shown as mean ± SEM. n.s., not significant; *P < 0.05; **P < 0.01; ***P < 0.001. (Two-way ANOVA multiple comparisons for **b**).

To rule out the possibility that an enhanced Th1 response might result from a higher viral load caused by the LTβR-Ig blockade, viral load in peripheral nervous tissues were detected on day 8 p.i. There was no significant difference in the peripheral viral load with or without LTβR-Ig treatment (Fig. 2a). The levels of the HSV-1 genome DNA in the DLN were also comparable between these two groups (Fig. 2b). Moreover, the Heat-iHSV infection model was employed to exclude the interference of viral replication. Though Heat-iHSV induced a weaker Th1 response than HSV-1 infection, LTβR signaling deficiency still resulted in an enhanced Th1 response in the Heat-iHSV infection (Fig. 2c).

To further investigate whether the enhanced Th1 response by LTα_1_β_2_-LTβR signaling deficiency is restricted to a specific viral infection, mice were immunized using OVA combined with CpG oligodeoxynucleotides (CpG-1826, a TLR9 agonist) as adjuvant (OVA-CpG). After OVA-CpG immunization, the LTβR signaling blockade again resulted in an enhanced anti-OVA Th1 response (Fig. 2d).

### T cell-derived LTα_1_β_2_ is required to limit Th1 response by modulating the lymphoid microenvironment

**Figure 3:**
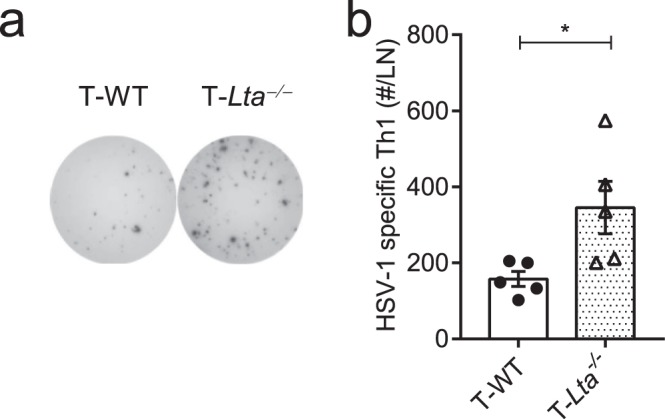
T cell-derived LT is required to limit Th1 response. T cells purified from WT and *Lta*^−/−^ mice were transferred to *Tcra*^−/−^ mice on the day before HSV-1 infection, abbreviated as T-WT and T-*Lta*^*−/−*^ mice; Th1 response in the T-cell conditional LTα-deficient mice on day 14 p.i. (n = 5/group), including immunospots (**a**) and absolute number of IFNγ-secreting CD4^+^ cells per LN (**b**). Data are representative of three independent experiments, shown as mean ± SEM. n.s., not significant; *P < 0.05; **P < 0.01; ***P < 0.001.

B cell-derived LT was proved essential in recruiting CXCR5^+^ T cells into the germinal center in *Heligmosomoides polygyrus* infection^26^. To determine if B cell-derived LTα_1_β_2_ could limit the Th1 response, splenic B cells purified from *Lta*^−/−^ *vs*. WT mice were adoptively transferred to the B-cell-deficient (μMT) mice before HSV-1 infection, abbreviated as B-*Lta*^−/−^ and B-WT mice. When analyzed on day 14 p.i., B-*Lta*^−/−^ mice generated a weaker Th1 response compared to the B-WT mice (Supplementary Fig. 2a–c). Given that LT is highly expressed on Th1 cells and was traditionally thought as a hallmark molecule of Th1 cells, we wondered if T cell-derived LTα_1_β_2_ would conversely limit an excessive Th1 response. To detect the role of LTα_1_β_2_ from T cells, splenic T cells from *Lta*^−/−^ *vs*. WT mice were adoptively transferred to the T-cell-deficient (*Tcra*^−/−^) mice before infection, abbreviated as T-*Lta*^−/−^ and T-WT mice. When analyzed on day 14 p.i., an enhanced Th1 response was generated in the T-*Lta*^−/−^ mice compared to the T-WT mice (Fig. 3a,b), suggesting that LTα_1_β_2_ from T cells limits the Th1 response in HSV-1 infection.

To further determine whether T cell-derived LTα_1_β_2_ limits the Th1-biased differentiation in an intracellular manner or indirectly by modulating the lymphoid environment via LTβR activation, mixed T cells from WT (CD45.1^+^) and *Lta*^−/−^ (CD45.1^−^) mice (1:1) were adoptively transferred to *Tcra*^−/−^ mice. After HSV-1 infection, there were no significant difference between the percentages of WT (CD45.1^+^) and *Lta*^−/−^ (CD45.1^−^) CD4^+^ cells in the LN of the recipient *Tcra*^−/−^ mice. Percentages of CD4^+^Tbet^+^ cells in WT (CD45.1^+^) *vs. Lta*^−/−^ (CD45.1^−^) CD4^+^ T cells were also comparable. *Lta*-deficient T cells differentiated in almost the same way as wild-type T cells when situated in the same environment (Supplementary Fig. 3a-c). We repeated this mixed-transferring experiment by transferring mixed WT-OT-II (Thy1.1^−^) T cells and *Lta*^−/−^-OT-II T (Thy1.1^+^) cells (1:1) to *Tcra*^−/−^ mice, and the recipient mice were immunized with OVA-CpG. The OT-II T cell differentiation again showed no difference between *Lta*^−/−^ and WT donor T cells in the recipient mice on day 10 post-immunization (Supplementary Fig. 3d–f). These indicate that the LTα_1_β_2_ deficiency in T cells does not intrinsically enhance the activation of Th1 cells. The fact that LTα_1_β_2_ from WT-T cells are sufficient to regulate a normal Th1 differentiation of *Lta*^−/−^ donor T cells actually suggests that T cell-derived LTα_1_β_2_ regulates the Th1 differentiation by modulating the lymphoid microenvironment.

### Blocking the LTα_1_β_2_-LTβR signaling enhances the Th1 response through over-production of IL-12

**Figure 4:**
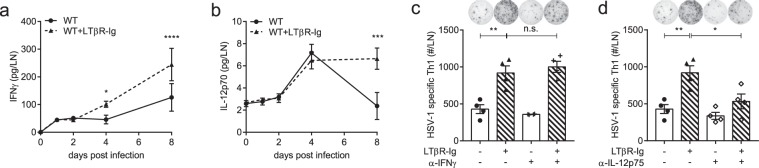
Blocking LT-LTβR signaling enhances Th1 response by over-production of IL-12. (**a**) IFNγ secretion in the popliteal LN from WT mice (solid line) and LTβR-Ig-treated mice (dotted line) detected by CBA test (n = 5/group). (**b)** IL-12 secretion in the popliteal LN from WT mice (solid line) and LTβR-Ig-treated mice (dotted line) detected by CBA test (n = 5/group). (**c)** Anti-IFNγ (clone XMG1.2, 500 μg/mouse) was i.p. administered every third day from the day before HSV-1 infection. Th1 response was detected on day 14 p.i. by Elispot (n = 4/group). Immunospots and absolute numbers of IFNγ-secreting CD4^+^ cells per LN are shown. (**d)** Anti-IL-12p75 (clone R5-9A2, 500 μg/mouse) was i.p. injected every third day from the day before HSV-1 infection. Th1 response was detected on day 14 p.i. by Elispot (n = 4/group). Immunospots and absolute numbers of IFNγ-secreting CD4^+^ cells per LN are shown. Data are representative of three independent experiments, shown as mean ± SEM. n.s., not significant; *P < 0.05; **P < 0.01; ***P < 0.001. (Two-way ANOVA multiple comparisons for **a,b**).

Based on the above, we tried to discover the potential Th1 promoting factors in the LTα_1_β_2_-LTβR signaling deficient lymphoid microenvironment. Increased IFNγ secretion was observed in the popliteal LN in LTβR-Ig-treated mice from day 2 post HSV-1 infection (Fig. 4a). Release of IL-12 was also maintained at a high level after day 4 p.i. under the LTβR-Ig blockade (Fig. 4b). As previously reported, IL-12 induces expression of both T-bet and Bcl6 via the transcription factor STAT4. During the transitional stage of both phenotypes, T-bet will finally promote a full Th1 polarization^27,28^. IFNγ was also reported to support the Th1 polarization in an autocrine manner^29^. To test if the LTβR-Ig enhanced Th1 response would be repressed by blocking the potential IL-12 or IFNγ pathways, neutralizing antibodies for the candidate Th1-promoting cytokines were employed. After systemic administration of the neutralizing antibodies every three days for three times in total, the enhanced Th1 response induced by the LTβR-Ig blockade was kept enhanced under the neutralization of IFNγ (Fig. 4c), but reversed by neutralizing IL-12 (Fig. 4d). These results indicate that the LTβR-Ig induces an over-production of IL-12 which promotes an excessive Th1 response.

### LTα_1_β_2_-LTβR signaling limits the Th1 response by controlling infiltration of monocytes and monocyte-derived DCs

**Figure 5:**
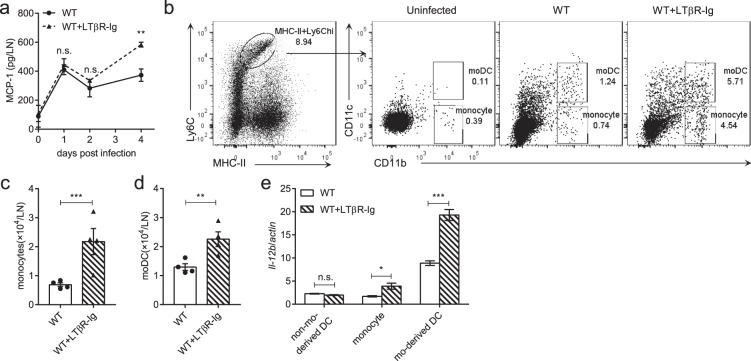
Blocking the LT-LTβR signaling promotes infiltration of monocytes and monocyte-derived DCs in the draining lymph node. (**a**) MCP-1 secretion in the popliteal LN from WT mice (solid line) and LTβR-Ig-treated mice (dotted line) detected by CBA test (n = 5/group). (**b)** Gating strategy of monocytes and moDCs, shown by representative dot plots from day 4 p.i. (**c**) Absolute number of monocytes in the popliteal LNs (n = 4/group). (**d)** Absolute number of moDCs in the popliteal LNs (n = 4/group). (**e)** Monocytes, moDCs and non-monocyte-derived DCs (CD11c^+^CD11b^−^Ly6C^−^MHC-II^+^) were purified on day 4 p.i. Expression of IL-12 was analyzed by RT-PCR (n = 3/group). Data are representative of three independent experiments, shown as mean ± SEM. n.s., not significant; *P < 0.05; **P < 0.01; ***P < 0.001. (Two-way ANOVA multiple comparisons for (**a**); two-tailed paired Student’s *t* test for **c–e**).

**Figure 6:**
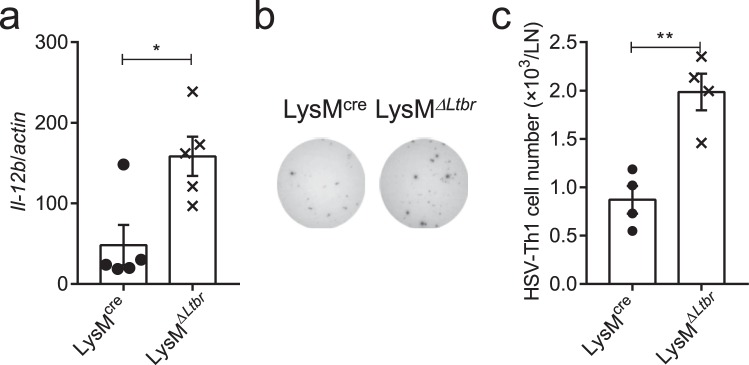
Blocking LT-LTβR signaling enhances Th1 response by up-regulating IL-12 expression from monocyte-derived cells. (**a)** Expression of IL-12 in the popliteal LN of LysM^*ΔLtbr*^ mice on day 4 p.i. (n = 5/group). (**b**,**c**) Th1 response of LysM^*ΔLtbr*^ mice on day 14 p.i. (n = 4/group). Immunospots (**b**), and absolute number of IFNγ-secreting CD4^+^ cells per LN (**c**). Data are representative of three independent experiments, shown as mean ± SEM. n.s., not significant; *P < 0.05; **P < 0.01; ***P < 0.001. (Two-tailed paired Student’s *t* test for **a,c**).

Who is the provider of the increased IL-12 during the LTα_1_β_2_-LTβR signaling deficiency? Release of MCP-1 was raised after the LTβR-Ig blockade in the popliteal LN early post HSV-1 infection (Fig. 5a). MCP-1, also known as CCL2, is a monocyte-recruiting chemokine^30^. Monocytes are known for secreting IL-12 during inflammation^31^, but monocyte-derived DCs (moDCs, CD11c^+^CD11b^+^Ly6C^hi^MHC-II^+^) and CD8α^+^ DCs are the major cellular source of IL-12 in *Toxoplasma gondii* cysts infection^32,33^. However, in the murine model of acute HSV-1 infection, CD8α^+^ DCs were considered critical for the CTL response^34–38^ while CD4^+^ T cells were proved to be primed by migratory APCs (no clarification of which subset of APCs)^38,39^. IL-12 production increased soon after HSV-1 infection and decreased after day 4 p.i. in wild-type mice. However, the LTβR-Ig blockade caused a prolonged IL-12 production after day 4 p.i., suggesting that the turning point of IL-12 provider cells happens around day 4 p.i. due to the LTβR-Ig treatment. With the LTβR-Ig administered, monocytes (CD11c^−^CD11b^+^Ly6C^hi^MHC-II^+^) and moDCs (CD11c^+^CD11b^+^Ly6C^hi^MHC-II^+^) were both found increased post HSV-1 infection in the LN (Fig. 5b–d). To further identify the main affected provider cells of IL-12, monocytes, moDCs and non-monocyte-derived DCs (CD11c^+^Ly6C^−^MHC-II^+^, used as control) were purified from the popliteal LN on day 4 p.i. and expression of IL-12 was analyzed by RT-PCR. Higher IL-12 expression was detected in moDCs and monocytes in the LTβR-Ig treated mice than control mice (Fig. 5e). Finally, LysM^*ΔLtbr*^ mice (hybridized from *Ltbr*^*flox/flox*^ × LysM^*cre*^) were utilized to confirm the function of LTβR signaling in monocytes and monocyte-derived cells *in vivo* (Supplementary Fig. 4a,b). Though no more infiltration of monocytes or moDCs were found in LysM^*ΔLtbr*^ mice post infection (Supplementary Fig. 4c,d), a significant up-regulation of IL-12 in the DLNs of the LysM^*ΔLtbr*^ mice was observed on day 4 p.i. (Fig. 6a). Further, LysM^*ΔLtbr*^ mice also generated an enhanced Th1 response in HSV-1 infection (Fig. 6b,c). LTβR activation of monocytes could inhibit the virus-induced up-regulation of IL-12 and limit the over-active Th1 response. Therefore, the accumulation of monocytes and moDCs with up-regulation of IL-12 promoted an enhanced Th1 response in the LTβR-Ig-blocked mice.

## Discussion

LT is expressed on naive T cells and constitutively expressed on Th1 but not Th2 cells. Similar to IFNγ^29^, LT is a key cytokine released from Th1 cells, which raises the possibility that LT may facilitate Th1 differentiation. In contrast, our data reveal that T cell-derived LTα_1_β_2_ suppresses the Th1 response during HSV-1 infection. LTβR-Ig treatment could also enhance the Th1 response to Heat-iHSV infection and OVA-CpG immunization. Thus, LTα_1_β_2_ helps to limit the Th1 response in a negative feedback manner, which is opposite to the positive feedback regulation of IFNγ. High expression of LTα_1_β_2_ on Th1 cells may counter-regulate the excessive Th1 differentiation and induce a balanced helper T cell response.

LTα_1_β_2_-LTβR signaling has been reported to regulate the helper T cell response. The LTβR-deficiency-caused defective Th1 response was thought to increase the susceptibility to *Citrobacter rodentium*^10^. However, several studies reveal that LT from type III innate lymphoid cells (ILC3) is essential for control of *Citrobacter rodentium*^40,41^. In *Leishmania major* infection, *Ltbr*^−/−^ and *Lta*^−/−^ mice both favored Th2 cells rather than Th1 cells, but the LN structure was then considered to be important^11^. It is possible that parasites induce strong Th2 dominant responses in the infected microenvironment. These suggest that Th1/Th2 biased responses regulated by the LTα_1_β_2_-LTβR signaling may differ depending on the pathogen-specific microenvironment to limit pathology and/or promote clearance of pathogens.

Recently, IFNγ-producing CD4^+^ and CD8^+^ T cells were reported to be increased in *Ltbr*^−/−^ → WT mice in the small intestinal lamina after oral infection of rotavirus^42^. In our study, no increase of IFNγ^+^CD8^+^ T cells were found after the LTβR-Ig blockade. Given that CD8α^+^ DCs have been considered critical for an optimal CTL response in HSV-1 infection^34–38^ and the increase of CD8α^+^ DCs in the draining LNs were found inhibited by the LTβR-Ig, anti-HSV-1 CTL responses were not enhanced. Together, the LTα_1_β_2_-LTβR signaling may play different roles in the Th1 and CTL responses in HSV-1 infection.

Altogether, this work uncovered a novel function of T cell-derived LTα_1_β_2_ in controlling the defined Th1 differentiation in the adaptive immune response, which enhances our understanding of LTβR-dependent Th1-induced autoimmune or inflammatory diseases. Targeting the LTβR-induced pathway may provide insights for Th1-oriented vaccine designs and treatments of inflammatory diseases.

## Electronic supplementary material

Supplementary figures
