# Supplementary material for: T cell-derived lymphotoxin limits Th1 response during HSV-1 infection
Source: Sci Rep. 2018 Dec 7;8:17727. doi: 10.1038/s41598-018-36012-z (PMC6286317; doi:10.1038/s41598-018-36012-z)
Supplement: Supplementary file 1 — Supplementary figures [file 41598_2018_36012_MOESM1_ESM.doc]

**T cell-derived lymphotoxin limits Th1 response during HSV-1 infection**

Kaiting Yang^1, 2^, Yong Liang^1^, Zhichen Sun^1, 2^, Longchao Liu^3^, Jing Liao^1^, Hairong Xu^1^, Mingzhao Zhu^1^, Yang-Xin Fu^3^, * and Hua Peng^1^, *

^1^Key Laboratory of Infection and Immunity, Institute of Biophysics, Chinese Academy of Sciences, Beijing 100101, China;

^2^University of Chinese Academy of Sciences, Beijing 100049, China;

^3^Department of Pathology, University of Texas Southwestern Medical Center, Dallas, TX 75390, USA

*** Corresponding author:**

Hua Peng, Mailbox 112, 15 Datun Rd, Chaoyang District, Beijing 100101, China. Tel: +86-010-64881152. E-mail address: [hpeng@moon.ibp.ac.cn](mailto:hpeng@moon.ibp.ac.cn)

Yang-Xin Fu, University of Texas Southwestern Medical Center, 6000 Harry Hines Boulevard, Dallas, TX 75390. E-mail address: [yang-xin.fu@utsouthwestern.edu](mailto:yang-xin.fu@utsouthwestern.edu)

**Supplementary figures**

**
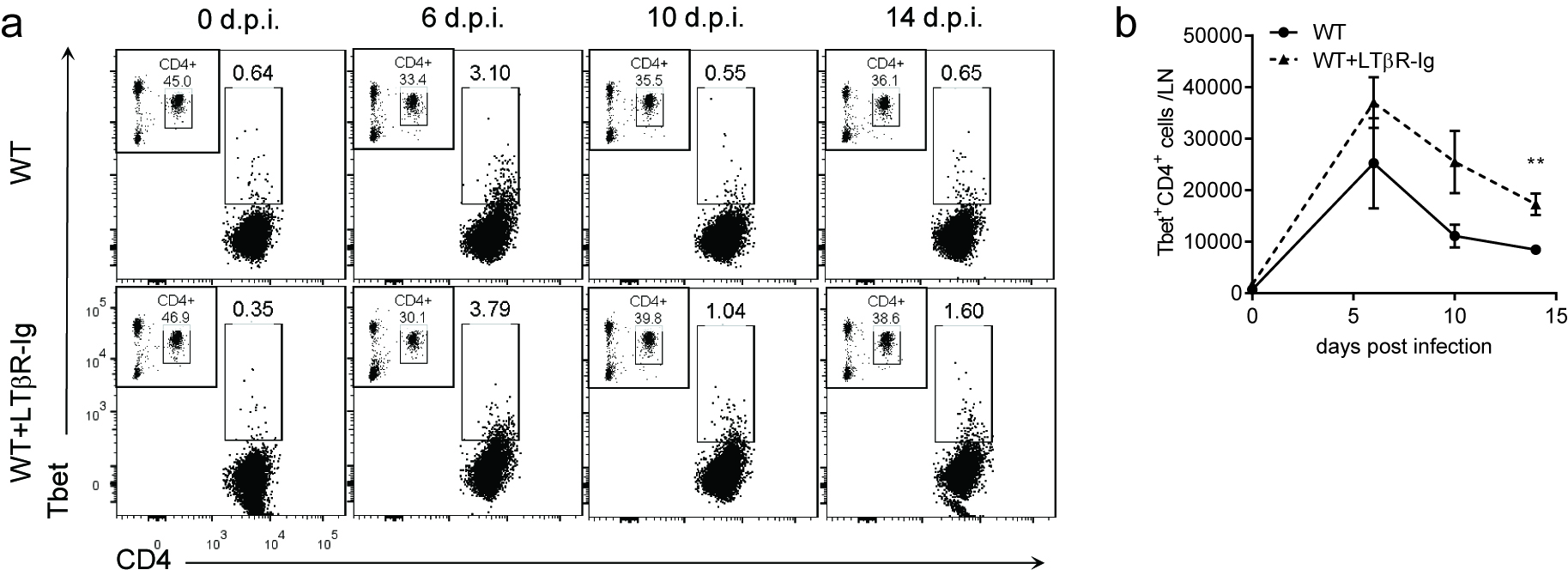
**

**Supplementary Figure 1. LTβR-Ig treatment enhances the HSV-1-specific Th1 response**

**a.** WT and LTβR-Ig treated C57BL/6 mice were infected by HSV-1 (5×10^7^ pfu/50μl). Representative dot plots gated from CD4^+^ T cells illustrate the percentages of CD4^+^Tbet^+^ cells in CD4^+^ T cells from the draining LNs (pooled from the ipsilateral popliteal LN and inguinal LN, with parent gating of CD4^+^ T cells shown in small window). (n=5/group). **b.** Numbers of CD4^+^Tbet^+^ cells in total cells in the draining LNs (popliteal LN and inguinal LN) from WT mice (solid line) and LTβR-Ig-treated mice (dotted line) (n=5/group). Data are representative of three independent experiments, shown as mean ± SEM. **P* < 0.05; ***P* < 0.01, and ****P* < 0.001 (Two-way ANOVA multiple comparisons for **b**).


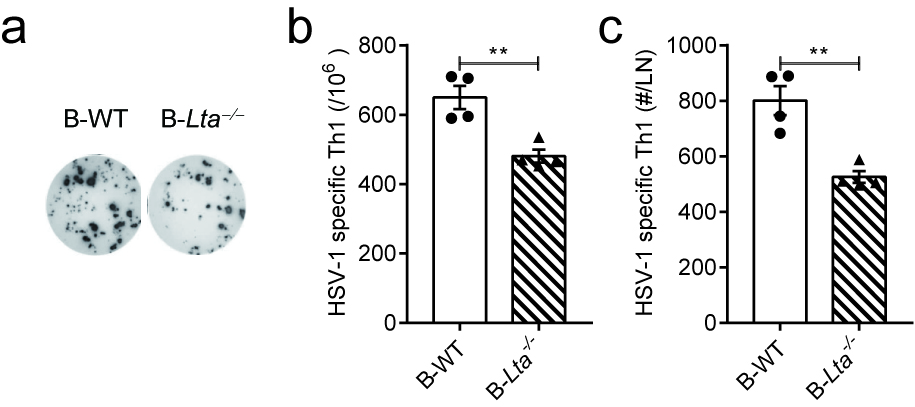


**Supplementary Figure 2. LT from B cells has no restriction on the Th1 response**

B cells purified from WT and *Lta*^−/−^ mice were transferred to μMT mice on the day before HSV-1 infection, abbreviated as B-WT and B-*Lta^−/−^* mice. **a-c.** Th1 response in the B-cell conditional LTα-deficient mice on day 14 p.i. (n=4/group), including immunospots (**a**), percentages (**b**) and absolute number (**c**) of IFNγ-secreting CD4^+^ cells per LN. Representative of three independent experiments. Data are shown as mean ± SEM. **P* < 0.05; ***P* < 0.01, and ****P* < 0.001 (two-tailed unpaired Student’s *t*-test).


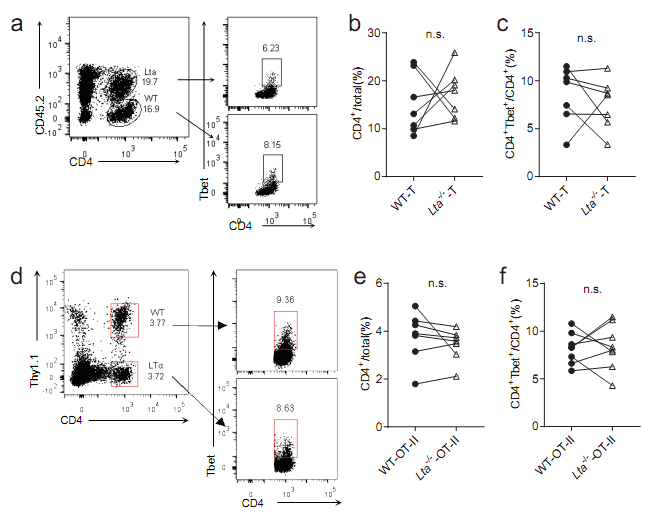


**Supplementary Figure 3. T cell-derived LT limits the Th1 differentiation in an exogenous manner**

**a-c.** Mixed T cells were transferred from WT *vs.* *Lta*^−/−^ mice (1:1) to *Tcra*^−/−^ mice, and the recipient mice were infected with 5×10^7^ pfu of HSV-1; T helper cell differentiation was analyzed on day 14 p.i. by FACS. Representative dot plots shown in **a**. Percentages of WT CD4^+^ T cells or *LTa^−/−^* CD4^+^ T cells in total cells (**b**). Percentages of CD4^+^Tbet^+^ cells in CD4^+^ T cells (**c**) (n=7/group). **d-f.** Mixed OT-II T cells were transferred from WT-OT-II (Thy1.1^−^) *vs. Lta*^−/−^-OT-II T (Thy1.1^+^) mice (1:1) to *Tcra*^−/−^ mice, and the recipient mice were immunized with OVA-CpG (100μg OVA and 50μg CpG); Representative dot plots showing gating strategy of percentages of CD4^+^Tbet^+^ cells in CD4^+^ T cells from the mixed-OT-II-T-cell transplantation model, analyzed on day 10 p.i. (**d**). Percentages of WT-OT-II (Thy1.1^−^) CD4^+^ T cells or *LTa^−/−^*-OT-II T (Thy1.1^+^) CD4^+^ T cells in total cells (**e**). Percentages of CD4^+^Tbet^+^ cells in CD4^+^ T cells (**f**) (n=7/group). Representative of two independent experiments. Data are shown as individual, two-tailed paired Student’s *t*-test. n.s. not significant.


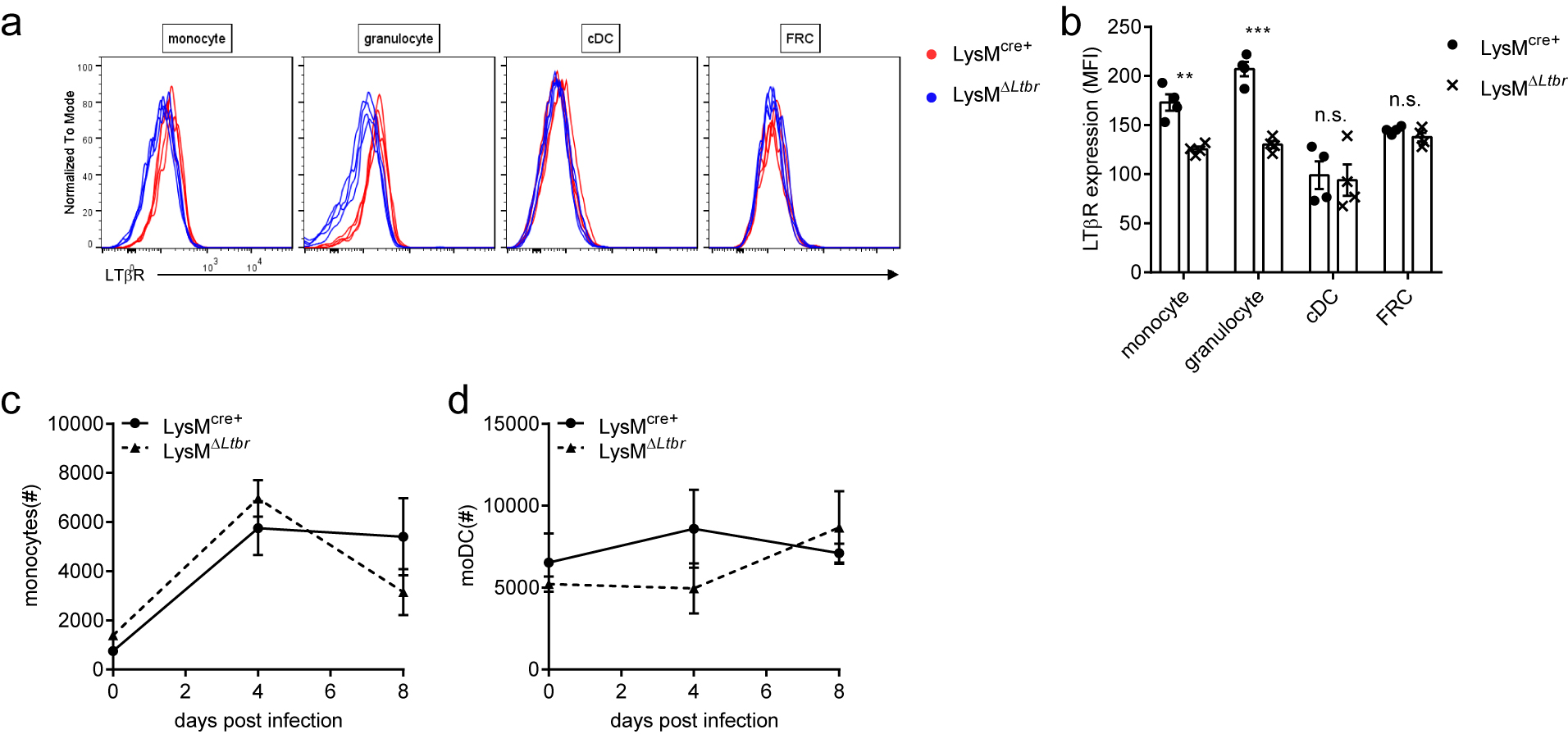


**Supplementary Figure 4. Infiltration of monocytes and monocyte-derived DCs in LysM*^ΔLtbr^* mice**

**a-b.** Expression of LTβR of cells from the LysM*^ΔLtbr^* mice (**a**, blue lines stand for the LysM*^ΔLtbr^* mice and red lines stand for control mice; **b**, statistical result of **a**, two-tailed unpaired Student’s *t*-test). **c.** Numbers of monocytes from the LysM*^ΔLtbr^* mice and control mice post HSV-1 infection (5×10^7^ pfu, n=4/group). **d.** Numbers of monocyte-derived DCs from the LysM*^ΔLtbr^* mice and control mice post HSV-1 infection (5×10^7^ pfu, n=4/group). Representative of two independent experiments. Data are shown as mean ± SEM. **P* < 0.05; ***P* < 0.01, and ****P* < 0.001.
